# “Wish You Were Here”: Examining Characteristics, Outcomes, and Statistical Solutions for Missing Cases in Web-Based Psychotherapeutic Trials

**DOI:** 10.2196/mental.8363

**Published:** 2018-04-19

**Authors:** Eyal Karin, Blake F Dear, Gillian Z Heller, Monique F Crane, Nickolai Titov

**Affiliations:** ^1^ eCentreClinic Department of Psychology Macquarie University Sydney Australia; ^2^ Mindspot Clinic Department of Psychology Macquarie University Sydney Australia; ^3^ Department of Statistics Macquarie University Sydney Australia; ^4^ Department of Psychology Macquarie University Sydney Australia

**Keywords:** psychotherapy, treatment adherence and compliance, missing data, treatment efficacy, statistical bias

## Abstract

**Background:**

Missing cases following treatment are common in Web-based psychotherapy trials. Without the ability to directly measure and evaluate the outcomes for missing cases, the ability to measure and evaluate the effects of treatment is challenging. Although common, little is known about the characteristics of Web-based psychotherapy participants who present as missing cases, their likely clinical outcomes, or the suitability of different statistical assumptions that can characterize missing cases.

**Objective:**

Using a large sample of individuals who underwent Web-based psychotherapy for depressive symptoms (n=820), the aim of this study was to explore the characteristics of cases who present as missing cases at posttreatment (n=138), their likely treatment outcomes, and compare between statistical methods for replacing their missing data.

**Methods:**

First, common participant and treatment features were tested through binary logistic regression models, evaluating the ability to predict missing cases. Second, the same variables were screened for their ability to increase or impede the rate symptom change that was observed following treatment. Third, using recontacted cases at 3-month follow-up to proximally represent missing cases outcomes following treatment, various simulated replacement scores were compared and evaluated against observed clinical follow-up scores.

**Results:**

Missing cases were dominantly predicted by lower treatment adherence and increased symptoms at pretreatment. Statistical methods that ignored these characteristics can overlook an important clinical phenomenon and consequently produce inaccurate replacement outcomes, with symptoms estimates that can swing from −32% to 70% from the observed outcomes of recontacted cases. In contrast, longitudinal statistical methods that adjusted their estimates for missing cases outcomes by treatment adherence rates and baseline symptoms scores resulted in minimal measurement bias (<8%).

**Conclusions:**

Certain variables can characterize and predict missing cases likelihood and jointly predict lesser clinical improvement. Under such circumstances, individuals with potentially worst off treatment outcomes can become concealed, and failure to adjust for this can lead to substantial clinical measurement bias. Together, this preliminary research suggests that missing cases in Web-based psychotherapeutic interventions may not occur as random events and can be systematically predicted. Critically, at the same time, missing cases may experience outcomes that are distinct and important for a complete understanding of the treatment effect.

## Introduction

### Background

Missing cases are often encountered in Web-based psychotherapeutic trials, with the likely frequency of participants to become absent from posttreatment surveys ranging from 1 in every 5, to 1 in every 3 patients [1,2]. Missing cases present a significant challenge to the accuracy of results by reducing the sample size and the statistical power available to estimate the effects of treatment [3]. Furthermore, missing cases can produce measurement bias by systematically concealing important clinical information such as the experience of negative outcomes in treatment.

Although multiple definitions of missing cases are possible (eg, unit, item) [4], this paper will consider missing cases as those individuals who conceal their treatment outcomes as absent cases at the point of posttreatment surveys. Without any information about the outcomes of missing cases, the challenge that these cases pose is that the clinical effect itself cannot be completely understood [3].

The problems associated with missing data are well recognized in the clinical literature, and reflecting this, requirements to account for missing cases are embedded in leading guidelines such as the Consolidated Standards of Reporting Trials statement [5] and other methodological guidelines [6-9]. Such guidelines require clinical researchers to make estimates about the treatment outcomes for missing cases and incorporate these estimates in the measurement and evaluation of treatment effects [7]. The statistical methods employed to account for missing cases’ outcomes typically attempt to mimic the remaining observed cases and simulate replacement treatment outcomes [6]. Examples of such statistical methods include model-based imputations and multiple imputations [9,10]. These statistical methods aim to resolve both issues of reduced sample size and potential measurement bias associated with overlooking missing cases outcomes [6,9,11].

When attempting to approximate and replace missing cases outcomes, statistical and methodological guidelines first advise that research explore for evidence about the characteristics and likely outcomes of missing cases. This is a first and pivotal step in the process of handling missing cases, which can lead to a more educated guess about the kind of clinical outcomes missing cases that would have likely occurred [6,9,12,13]. In more statistical terms, researchers are required to make an informed assumption about the unknown outcomes for missing cases and effectively decide whether missing cases are a distinct subgroup with distinct and important outcomes or a random and ignorable extension of the whole sample [6,13]. It is also important to note that any characterization of missing cases and the replacement of their outcomes is made under one of three possible assumptions [8]. First, the assumption that missing cases and their outcomes are comparable with the characteristics and outcomes of the overall reaming sample is named the missing completely at random (MCAR) assumption. Similarly, the assumption when missing cases show some distinct characteristics but are assumed to be comparable in outcomes with a similar subgroup of remaining cases (stratified subgroup) is named the missing at random (MAR) assumption. Alternatively, if missing cases are assumed to have characteristics and outcomes that are not comparable to any subset of the reaming cases, the assumption of missing not at random (MNAR) is made.

Notwithstanding the range of statistical solutions [14], guidelines [15], and theoretical discussions [4] about missing cases in psychotherapy or Web-based psychotherapy, questions remain about the characteristics and solutions that could be applied to missing cases following treatment.

The first question regards the characteristics of missing cases and the ability to identify any systematic predictors of missingness. Currently, no concerted empirical studies are available to identify and assess those participant characteristics that are likely to increase the likelihood of becoming missing at posttreatment. As separate from the dropout and treatment adherence literature [2,16-18], factors that predict whether a case will become missing have not been explored within large-scale psychotherapeutic studies; although it is conceivable that these overlap [19].

A second related question concerns the ability to identify variables that describe why missing cases occurred and at the same time give reason to suspect that the outcomes for missing cases are distinct from the overall sample [2,6,7]. For example, if missing cases were characterized by lower treatment adherence, the treatment outcomes of missing cases should also be impacted by lower treatment dosage. This hypothetical example illustrates a scenario where individuals with poorer fit to treatment remove themselves from treatment, conceal their outcomes as missing cases, and leave the evaluation of treatment results to be determined by a margin of people to whom the treatment appeals. In these circumstances, recognizing the role of predictors, such as treatment adherence, is critical for the ability to detect both the increased risk of cases to become missing, as well as for the ability to approximate accurate replacement outcomes for such cases [6,9,10,15].

A third consequent unanswered question concerns the relative accuracy of replacing missing psychotherapy cases under different statistical missing cases strategies and assumptions. Without studies that investigate missing cases and their likely outcomes in the context of psychotherapy, Web-based psychotherapy, or other similar clinical fields, uncertainty remains about the ability to replace and handle missing cases [9]. To explore the suitability of different missing cases solutions, comprehensive clinical research is required that can compare simulated outcomes for missing cases against a proximal outcome of missing cases. Currently, no solutions are available within the Web-based psychotherapy literature to suggest a benchmark for proximally measuring the outcomes for missing cases. As a consequence, no evidence is currently available to support or refute the suitability of any type of statistical strategy or quantify the implications missing cases have for the estimation of treatment effects.

### This Study

The primary aim of this study was to empirically explore evidence from a large naturalistic Web-based psychotherapy sample and provide evidence toward three interrelated questions about missing cases. Specifically, this study sought to (1) identify the characteristics and dominant predictors of missing cases, (2) identify predictors that may have joint influence on likelihood of missing cases and clinical outcomes, and (3) identify a suitable clinical measurement benchmark that can then be used to test the accuracy and suitability of different statistical replacements strategies.

Three hypotheses were made about the characteristics of missing cases and the ability to approximate their outcomes. Consistent with previous theoretical discussions of missing cases in psychotherapy [2,19] and clinical trials [8,20], it was hypothesized that missing cases do not occur as a random event (H1), and participant and treatment features such as treatment adherence would predict the likelihood of participants to present as missing cases following treatment. Second, consistent with the dropout and adherence literature [2,19], it was hypothesized that cases that became missing during posttreatment would be characterized with lower treatment adherence (H2). Third, consistent with statistical guidelines [9,15], it was hypothesized that the replacement of clinical outcomes for missing cases would be made with minimal measurement bias, on the condition of adjusting for key predictors (H3).

## Methods

### The Sample

This study employed clinical data from three large randomized controlled trials (RCTs; n *=* 820) investigating the efficacy of Web-based cognitive behavioral therapy (CBT) interventions for reducing symptoms of anxiety and depression [21-23]. These trials employed a similar recruitment methodology and treatment procedures under the Macquarie University Web-based Model (MUM) [24], involving the weekly delivery of Web-based materials organized into psychotherapeutic lessons, together with notifications, emails, and survey reminders over a period of 8 weeks. Telephone contact by a trained clinician was attempted in combination with reminder emails in efforts to engage participants and increase survey participation following treatment. This contact protocol was uniformly applied before treatment, at the end of treatment, and at the point of 3-month follow-up to facilitate participant engagement and adherence.

To be included in these trials, participants were selected on the basis of (1) Demonstrating at least minimal symptoms of anxiety or depression, as determined by the presence of at least mild symptoms of depression or anxiety (a minimum score ≥5 on either the Patient Health Questionnaire 9-item, PHQ-9 [25]; or the Generalized Anxiety Disorder Scale 7-item, GAD-7 [26]); (2) Being over the age of 18 years; (3) Being an Australian resident; and (4) Having Internet access for the period of the trial. In addition, applicants who reported a score of 3 (considered severe) on item 9 of the PHQ-9 measuring suicide-risk were referred to another service.

In combination, these trials represent a random intake of adults seeking treatment for symptoms of depression and anxiety over a period of 2 years within the eCentreClinic [27]. The demographic and symptom characteristics of the participating sample are shown in Table 1.

It is important to note that Web-based psychotherapy data can present a unique opportunity for investigating missing cases and their trajectories in treatment. The standardization of treatment engagement and materials can be considered to reduce the outcome measurement variance associated with treatment delivery. With reduced treatment related variance, the individual’s response to treatment remains the main source of statistical variation. In more statistical terms, this sample represents a unique opportunity to measure missing cases influences and outcomes with increased internal validity and within a large sample, enabling a robust statistical testing of the first and second hypotheses. In addition, this sample collates a unique subsample of individuals who are missing at posttreatment but are successfully recontacted during a clinical follow-up, enabling a niche subsample that can be used to test the third hypothesis.

### Measures

The primary outcome measure for this study was the PHQ-9, a quantitative measure of depressive symptoms [25]. The PHQ-9 is widely used in psychotherapy and Web-based psychotherapy, is sensitive to the presence and severity of depressive symptoms, and is illustrative of high internal consistency [24,28]. Total scores range from 0 to 27, and the scale comprises 9 items, each offering four responses ranging from 0 to 3. Total scores are clinically interpreted: no depression (total score: 0-4), mild depression (total score: 5-9), moderate depression (total score: 10-14), moderately severe depression (total score: 15-19), and very severe depression (total scores: 20-27). PHQ-9 baseline symptom of the sample are presented in Table 1.

The PHQ-9 scale was administered to measure symptoms at pretreatment (baseline), posttreatment, and again 3 months after the completing of treatment. The original trials comprising the dataset all demonstrated significant and similar average symptom reductions from baseline to posttreatment (46%-53%), which were maintained at 3-month follow-up (50%-53%).

Comorbidity, demographic measures, and treatment adherance were also included as independent variables, aiming to predict missing cases and their clinical trajectories through treatment.

#### Comorbidity

Participants were defined as having comorbidity if they demonstrated scores of anxiety and depression above a predetermined clinical threshold (GAD-7≥8 and PHQ-9≥10 at baseline; GAD-7 [25]; PHQ-9 [29]).

**Table 1:** Demographic and clinical sample characteristics. GAD-7: Generalized Anxiety Disorder Scale 7-item; N/A: not applicable; PHQ-9: Patient Health Questionnaire 9-item.

Characteristics		Total sample collated, value (n=820)		Completers^a^, value (n=682)		Missing cases^b^, value (n=138)		Recontacted cases^c^, value (n=55)	
**Gender, n (%)**									
	Female		606 (73.9)		465 (75.2)		95 (68.8)		39 (71)
	Male		214 (26.1)		153 (24.8)		43 (31.2)		16 (29)
Age, mean (SD)		43.2 (11.1)		44.1 (11.4)		40.4 (11.1)		38.1 (11.4)	
**Treatment adherence, n (%)**									
	Completed (1 of 5)		65 (7.9)		9 (1.5)		49 (35.5)		14 (25)
	Completed (2 of 5)		53 (6.5)		26 (4.2)		24 (17.4)		6 (11)
	Completed (3 of 5)		76 (9.3)		39 (6.3)		23 (16.7)		13 (24)
	Completed (4 of 5)		145 (17.7)		101 (16.3)		22 (15.9)		10 (18)
	Completed all modules		481 (58.7)		443 (71.7)		20 (14.5)		12 (22)
**Relationship status, n (%)**									
	Otherwise		306 (37.3)		215 (34.8)		62 (44.9)		23 (42)
	In a relationship		514 (62.7)		403 (65.2)		76 (55.1)		32 (58)
**Education, n (%)**									
	Non-tertiary		356 (43.4)		254 (41.1)		71 (51.4)		26 (47)
	Tertiary		464 (56.6)		364 (58.9)		67 (48.6)		29 (53)
GAD-7 baseline, mean (SD)		11.3 (4.6)		11.0 (4.6)		12.0 (4.6)		11.9 (4.8)	
PHQ-9 baseline, mean (SD)		12.3 (4.7)		11.9 (4.8)		13.9 (4.4)		13.7 (4.5)	
**Comorbidity, n (%)**									
	None		345 (42.1)		277 (44.8)		44 (31.9)		17 (31)
	Comorbid		475 (57.9)		341 (55.2)		94 (68.1)		38 (69)
Missing at posttreatment, n (%)		138 (16.8)		N/A^d^		N/A		55 (40)	
Missing at follow-up, n (%)		147 (17.9)		N/A		N/A		N/A	

^a^Individuals that completed all surveys.

^b^Individuals with any missing posttreatment data.

^c^Individuals recontacted at 3-month follow-up (n=55).

^d^N/A: not applicable.

#### Demographic Measures

Age in years at the start of treatment, relationship status, pretreatment symptom scores, pretreatment anxiety scores, and education background were considered. The categories created to measure levels of education, relationship status, treatment adherence, and gender are presented in Table 1.

#### Treatment Adherence

Under the MUM Internet CBT (iCBT) model, treatment material was organized through five Web-based lessons over a period of 8 weeks. Each lesson comprised introductory CBT explanations, homework assignments, cases stories, and other materials [24]. Participants were required to complete each of the five Web-based lessons in sequence to gain access to the subsequent lesson. Adherence to treatment was therefore measured in this study as the incremental indication that an individual has logged on to the assigned secured website and accessed the Web-based material as these were made available over time. In this way, treatment adherence was measured as the minimal but continued progression of participants through the intended course design.

### Recontacted Follow-Up Cases as a Proximal Outcome for Missing Cases at Posttreatment

A key subsample of interest in this study were those participants who presented as missing cases at posttreatment but recontacted at follow-up. In total, 83.2% of participants (682/820) completed the self-report symptom questionnaires at posttreatment. Out of those 138 participants who did not complete the posttreatment survey, 60.1% (83/138) also did not complete questionnaires at the 3-month follow-up. However, 40.0% (55/138) of participants who were missing at posttreatment were successfully surveyed through a 3-month clinical follow-up effort. These recontacted individuals were considered as cases who were partly missing at posttreatment, who would have been completely missing within study designs that followed a pre-post only protocol. Recontacted cases could be used as a proximal measurement of missing posttreatment outcomes, on the condition that recontacted cases show similarities to cases who were missing at both post and follow-up; as individuals belonging to a broader category of individuals with missing cases.

### Analytical Plan

Statistical analysis was conducted with three steps. The first step aimed to characterize missing cases by testing for significant predictors of missing cases (H1, H2). Initially, all possible predictors of missing cases were tested through separate logistic regression models. Within those logistic regression models, missing posttreatment cases versus nonmissing were the binary dependent variable. Following a series of univariate models, a stepwise model building analysis was attempted with the intention to identify a multivariate but parsimonious model of missing cases predictors. This was done by considering all possible predictors in a saturated binary logistic model, including treatment adherence, baseline depression score, baseline anxiety score, and demographic variables of gender, age, employment status, education status, and relationship status. Following, a stepwise variable selection strategy was taken, as outlined by Harrell [30], where predictors that increased the odds of becoming a missing case were retained in a final model. These remaining predictors were interpreted as dominant predictors that statistically characterize the features of missing cases. Each possible predictor of missing cases was assessed for statistical significance at an adjusted *P* value of .01 or less. In addition, the pseud- *R* squared, associated with each missing cases predictor was reported, aiming to convey the known, or model related, proportion of missing cases probability variance; with larger pseud- *R* squared indicating greater outcome predicative success, with a maximum of 1 [31]. In parallel to the prediction of missing cases, longitudinal models of symptom remission were conducted. These models intended to identify those participant characteristics that jointly predict missing cases and increased or decreased rate of symptom improvement following treatment. Longitudinal predictors of symptom change were examined with generalized estimating equation (GEE) models [32], as a series of separate univariate models. In combination, this step intended to test the ability of any one variable to predict missing cases likelihood, as well the outcomes those individuals were likely to experience at posttreatment.

In a second step, the 55 participants who were missing at posttreatment, but successfully recontacted at the 3-month follow-up, were also tested for their ability to represent missing cases who remained missing at both posttreatment and follow-up. The intention of this step was to suggest evidence that recontacted cases could be used as a proxy for missing posttreatment cases as a broader group. This was achieved by (1) Comparing the baseline symptom scores of cases with complete information (“completers”), missing cases at both time points (“completely missing cases”), and cases who are missing at post but are recontacted at 3-month follow-up (“recontacted cases”); (2) The characteristics of recontacted cases and completely missing cases were compared in a binary logistic regression seeking to test for differences between those recontacted cases and cases who were missing at both time points; and (3) To determine whether scores at 3-month follow-up could approximate posttreatment scores more broadly, a comparison between posttreatment and follow-up scores was conducted. In other words, testing whether missing cases who were recontacted at 3-month follow-up were likely to have similar treatment outcomes at posttreatment. Overall symptom change between post treatment and follow-up was tested with a longitudinal GEE model, testing for any additional symptom change between posttreatment and follow-up symptom outcomes.

In a third step, the third hypothesis was operationalized. This step compared simulated replacement scores, approximated by various adjusted models, against known outcome scores from recontacted cases. The aim of the third step was to quantify and test the relative accuracy of predicted replacement scores against known, proximal recontacted cases outcomes. Simulated follow-up scores were generated using longitudinal GEE and mixed models [33] as common longitudinal methods in clinical trials [34]. All models included a gamma scale, unstructured pattern of within subjects’ correlation over time, and log link function to account for positive skewness and proportional remitting symptoms from baseline [21-24].

Various simulated scores were evaluated as either overestimating, underestimating, or being equivalent to recontacted cases scores in accordance to the degree they predicted the observed outcomes of recontacted cases. Specifically, if the mean CI of the simulated symptom replacement scores included the mean symptom outcome of the recontacted cases, statistical equivalence was interpreted [35]. If the CI interval of the mean replacement scores would exclude the mean of the recontacted cases, the simulation models were considered to overestimate or underestimate the outcomes of missing cases.

Statistical analysis was conducted using Statistical Package for the Social Sciences (SPSS) [36] version 22 (IBM Corp).

## Results

### Step 1 (H1, H2)—Joint Predictors of Missing Cases and Clinical Outcomes

Results from the first step, testing for predictors of missing values at posttreatment through univariate and multiple logistic regression models, are presented in Table 2.

These results demonstrate that as separate univariate models, and as a multivariate model, the stepwise variable selection identified baseline depressive symptoms (Wald χ^2^_1_=152.4, *P*<.001) and treatment adherence (Wald χ^2^_4_=10.1, *P*<.01) were the dominant predictors of missing cases probability. Together, these variables predicted 40.3% of the probability variance (Nagelkerke pseudo R squared=0.403), with treatment adherence accounting for the majority of that variance as a single dominant predictor (39%).

The impact of increased baseline severity demonstrated that for every one additional unit on the PHQ-9 at baseline, the odds of a participant to become a missing posttreatment case increased relatively by 8.4% (1.5% as a relative risk). The predictor of treatment adherence demonstrated a strong but nonlinear predictor of missing cases probability. Specifically, participants who completed the entire program had only a 4% probability of becoming missing at posttreatment. In contrast, participants who completed only one lesson were over 70 times more likely to have missing posttreatment values relative to participants who attempted all five lessons (odds ratio=0.014).

An interaction between depressive baseline severity and treatment adherence was also explored and was found to be nonsignificant (Wald χ^2^_4_=3.0, *P*=.56). The nonsignificant interaction implies that baseline severity and treatment adherence were separate in their influences on missing cases.

Variables that influenced (moderated) the rate of symptom improvement were also tested. These analyses aimed to identify those participant characteristics that predicted the likelihood of an individual to become missing at posttreatment and at the same time, predict an individual’s clinical outcome. Each of the nine variables were examined for their ability to predict increased symptom reduction following treatment through the statistical testing of a time by covariate interaction term. These interaction coefficients are presented in Table 3.

**Table 2:** Logistical regression model testing for predictor of missing cases of posttreatment. GAD-7: Generalized Anxiety Disorder Scale 7-item; PHQ-9: Patient Health Questionnaire 9-item.

Predictors of missing values			Univariate models				Multivariate models (*P*=.05)^a^		
			*P*	Odds ratio	Percentage of missing cases^b^ (95% CI)	Variance explained, %	*P*	Percentage of missing cases^b^ (95% CI)	Variance explained, %
**Demographic**							—	—	
	Age (% per year)		<.001	0.97	−3 (−2 to −5]	3			
	**Gender**						—	—	
		Female	.14		16 (13-19)				
		Male		0.74	20 (15 to 20)	<1			
	**Relationship status**						—	—	
		In a relationship	.04		15 (12 to 18)				
		Otherwise		1.46	20 (16 to 25)	1			
	**Education level**						—	—	
		Tertiary education	.047		14 (12 to 18)				
		Otherwise		1.48	20 (16 to 24)	1			
**Initial severity** * *					0 (0 to 0)				
	Baseline anxiety symptoms (% per GAD-7 point)		.03	1.05	5 (0.5 to 9)	1	—	—	
	Baseline depression symptoms (% per PHQ-9 point)		<.001	1.09	9 (5 to 14)^c^	4	.002	8 (3 to 14)^c^	40
	Comorbidity at baseline: (PHQ-9≥10 and GAD-7≥8)		.01		20 (16 to 24)		—	—	
	None			0.59	13 (10 to 17)	2			
**Treatment adherence**									
	Completed all modules		<.001		4 (3 to 6)^c^	39		4 (3 to 6)^c^	40
	Completed (4 of 5)			4.12	15 (10 to 22)		<.001	14 (9 to 21)	
	Completed (3 of 5)			10	30 (21 to 41)		<.001	27 (18 to 38)	
	Completed (2 of 5)			19.08	45 (33 to 59)		<.001	42 (29 to 56)	
	Completed (1 of 5)			70.59	75 (64 to 84)		<.001	75 (63 to 84)	

^a^All models are based on a logistic regression model, including a log link function. Overall model accuracy for classification of missing values outcomes was 87.4%, with a specificity of 96.6% and sensitivity of 42%.

^b^Percentage of relative risk of an individual to become becoming missing at posttreatment.

^c^Relative odds of an individual to become a missing case with every additional unit increase.

**Table 3:** Association of predictor variables with clinical symptom change from baseline. GAD-7: Generalized Anxiety Disorder Scale 7-item; GEE: generalized estimating equation; PHQ-9: Patient Health Questionnaire 9-item.

Predictor of rate of clinical change			GEE^a^ univariate models			Mixed univariate models		
			Moderation of symptom change (Time×IV) at posttreatment			Moderation of symptom change (Time×IV) at posttreatment		
			*P*	Wald chi-square (degrees of freedom)	Percentage change^b^,^c^ (95% CI)	*P*	*F* statistic (degrees of freedom)	Percentage change^b^,^c^ (95% CI)
**Demographic**								
	Age (years, % per year)		.03	7.1 (1)	−1 (0 to −2)	.007	1.8 (1,1071)	<1 (<1 to <1)
	**Gender**							
		Female (versus male)	.43	1.7 (1)		.27	1.3 (1,1071)	
	**Relationship status**							
		In a relationship (versus otherwise)	.21	3.2 (1)		.22	1.5 (1,1071)	
	**Education level**							
		Tertiary (versus otherwise)	.17	3.5 (1)		.15	1.9 (1,1071)	
**Initial severity**								
	Baseline anxiety symptoms (% per GAD-7 point)		>.99	0.1 (1)		.98	<0.1 (1,1071)	
	Baseline depression symptoms (% per PHQ-9 point)		<.001	22.3 (1)	2 (1 to 3)	<.001	11.6 (1,1071)	2 (1 to 3)
	Comorbidity at baseline: (PHQ-9≥10 and GAD-7≥8)		.16	3.6 (1)		.10	2.3 (1,1071)	
	None							
**Treatment adherence**			<.001	39.0 (4)		<.001	3.6 (4,1071)	
	Completed all modules				49 (45 to 52)			40 (16 to 56)
	Completed (4 of 5)				40 (32 to 47)			29 (0 to 50)
	Completed (3 of 5)				46 (36 to 55)			26 (−7 to 49)
	Completed (2 of 5)				42 (27 to 53)			35 (2 to 57)
	Completed (1 of 5)				21 (−8 to 43)			19 (−11 to 41)

^a^All models are based on a GEE model of change over time, interacting with a covariate.

^b^Percentage indication of a change from baseline.

^c^Marginal means reported for predictors with statistical significance (*P*<.05).

From Table 3, treatment adherence, baseline symptom levels, and age significantly moderated rate of symptom improvement following therapy. Greater rates of symptom improvement were observed with higher levels of treatment adherence and higher baseline depression scores.

**Figure 1:**
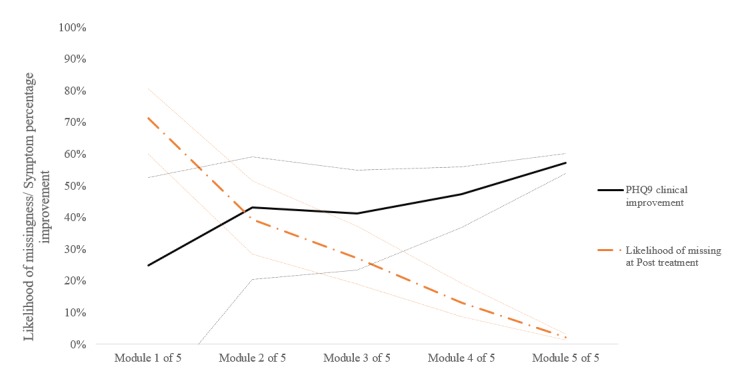
Treatment adherence (competition out of five modules) and the likelihood of missing cases or symptom improvement from pretreatment levels (%); dotted lines illustrate 95% CI of the estimate. PHQ-9: Patient Health Questionnaire 9-item.

**Figure 2:**
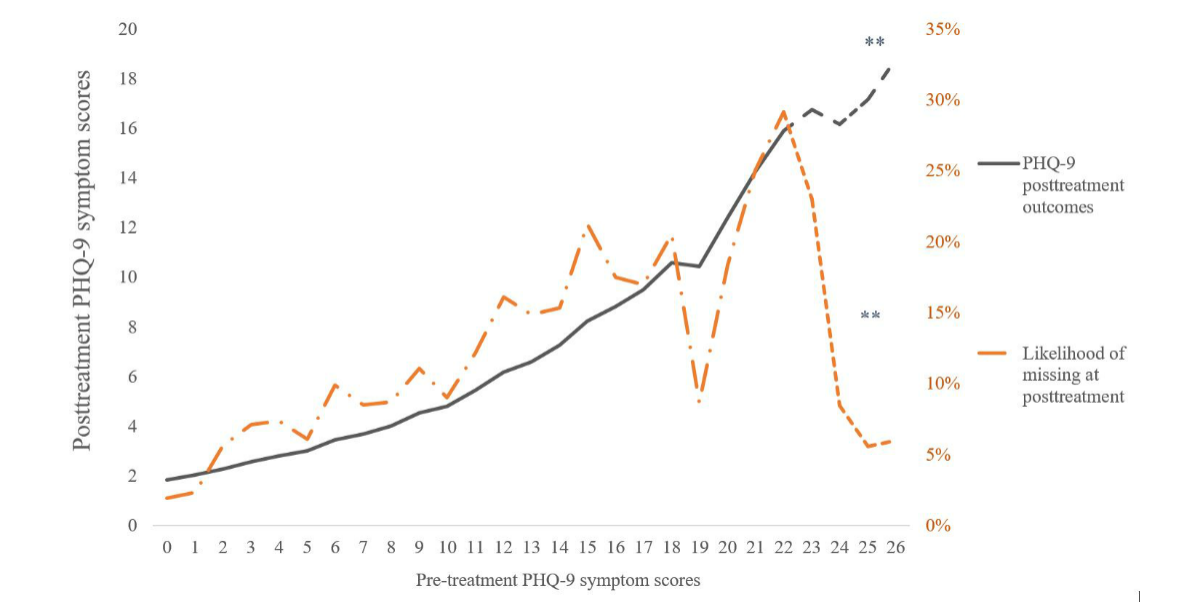
Pretreatment Patient Health Questionnaire 9-item (PHQ-9) symptoms influencing likelihood of missing cases or symptom outcomes. The **-dotted line implies a sample size of <10 participants from the sample of 820.

Taken together, the predictors of treatment adherence and baseline PHQ-9 symptoms demonstrated a joint association with both the rate of clinical improvement and the likelihood of missing data at posttreatment. The ability of treatment adherence and PHQ-9 baseline symptoms to influence both clinical outcomes and missing cases probability is graphically illustrated in Figure 1 (missing cases likelihood and symptom change trends associated with program adherence) and Figure 2 (missing cases likelihood and symptom outcome trends associated with baseline severity).

### Step 2—Testing Recontacted Cases as a Proxy of the Broader Group of Missing Cases

This step intended to establish evidence that recontacted cases at 3-month follow-up could be used as a proxy for the unknown outcomes of posttreatment missing cases. Initially, the baseline symptoms scores of the 3 missing cases subgroups were compared with a simple analysis of variance. A pairwise comparison of the PHQ-9 baseline symptom scores among the 3 groups indicated that participants who completed the surveys at both time points demonstrated overall lower PHQ-9 symptoms at baseline (PHQ-9 of 12.0; 95% CI 11.6-12.3) compared with recontacted cases (PHQ-9 of 13.7; 95% CI 12.5-15.0; *P*<0.001) and cases who were missing at posttreatment and 3-month follow-up (PHQ-9 of 13.6; 95% CI 12.6-14.1; *P*<0.001). However, participants who were recontacted at follow-up demonstrated equivalent symptom scores (*P*=0.54) to those participants who were completely missing. This finding indicated that missing cases and recontacted cases shared similarities as a group of individuals who present with missing cases.

A second analysis was conducted attempting to identify differences between those individuals who were missing cases and recontacted (55/138) and those individuals who were missing at posttreatment and follow-up (83/138). A logistic regression that specified recontacts and completely missing cases as its binary outcome was conducted. All possible predictors of missing cases were considered and assessed for statistical significance at a *P* value of .05 or less to account for the size of the subgroup (n=138). The resulting logistic regression models did not identify any one predictor that could explain the probability of missing or recontacted status.

A third longitudinal GEE analysis was conducted to corroborate that posttreatment and follow-up symptom scores were similar enough on average to be used interchangeably. Consistent with previous findings [23], a 45% reduction in symptoms was observed from baseline (PHQ-9 of 12.3 [95% CI 12.0-12.7]) to posttreatment (PHQ-9 of 6.4; 95% CI 6.0-6.8; Wald χ^2^_2_=572.1; *P<* 0.001), with only a smaller (>7%) but significant additional improvement (PHQ-9 of 5.9; 95% CI 5.6-6.3; Wald χ^2^_2_=6.4; *P<* 0.001) detected between posttreatment and follow-up time points.

Together, these 3 results illustrated that the recontacted follow-up cases of this study present as a close, albeit imperfect, proxy for the outcomes of the broader group of individuals with missing posttreatment cases.

### Step 3 (H3)—Using Recontacted Cases to Test the Accuracy of Simulated Replacement Score Under the Missing at Random, Missing Completely at Random, and Missing Not at Random Assumptions

In this step, the suitability of simulated replacement scores was explored by comparing the various predicted replacement scores against the known follow-up symptom outcome scores from recontacted individuals (Mean=8.11, 95% CI 6.53-10.07).

Table 6 presents the simulated mean PHQ-9 scores and CIs for replacement scores generated under different unadjusted and adjusted statistical models, as well as through the last observation carried forward (LOCF) and baseline observation carried forward (BOCF) methodology.

Table 6 illustrates those models that overlooked missing cases characteristics and did not adjust the approximation of missing cases; *underestimated* the symptom outcome scores of recontacted cases by as much as 30%. Similarly, replacement methods such as LOCF and BOCF both produced *significantly higher* estimates of symptom outcomes following treatment (24% and 69%, respectively).

Table 7 presents the mean and CIs generated through models that conditionally adjusted their estimation of missing cases outcomes. The approximated scores generated from each model are presented in Table 7 in descending order of accuracy; relative to the actual scores observed for recontacted cases. These results demonstrated that from the range adjusted models, models that included either treatment adherence or baseline severity in the prediction of outcomes could be interpreted as statistically equivalent to actual scores observed at 3-month follow-up. Specifically, both the GEE model and mixed model that adjust their estimates for treatment adherence and baseline severity resulted in the minimal approximation error (8%) relatively to the observed mean from actual outcomes.

Together, given some of the adjusted models were able to capture close approximations of the observed recontacted cases outcomes, the assumption of that missing cases cannot be conditionally compared with the remaining cases was refuted (MNAR).

**Table 6:** Depression (Patient Health Questionnaire 9-item, PHQ-9) simulate (approximated) replacement scores—unadjusted (missing completely at random, MCAR) models, last observation carried forward (LOCF), and baseline observation carried forward (BOCF). GEE: generalized estimating equation; N/A: not applicable.

Source of PHQ-9 estimates	Mean (95% CI)	Relative percentage accuracy from recontacted cases (95% CI)	Conclusion drawn about accuracy^a^
Recontacted cases	8.11 (6.53-10.07)	N/A	
BOCF	13.75 (12.57-15.03)	69 (55-85)	Significant overestimation
LOCF	9.96 (8.65-11.48)	24 (7-42)	Significant overestimation
MCAR (GEE)	5.93 (5.58-6.3)	−27 (−22 to −31)	Significant underestimation
MCAR (mixed)	5.96 (5.62-6.34)	−26 (−14 to −37)	Significant underestimation

^a^Relative accuracy from observed recontacted cases following a clinical follow-up.

**Table 7:** Depression (Patient Health Questionnaire 9-item; PHQ-9) simulate (approximated) replacement scores from various adjusted models. GAD-7: Generalized Anxiety Disorder Scale 7-item; GEE: generalized estimating equation; MAR: missing at random; N/A: not applicable.

Source of PHQ-9 estimates	Mean score (95% CI)	Relative percentage accuracy from recontacted cases (95% CI)	Conclusion drawn about accuracy^a^
Observed symptom score from recontacted cases	8.11 (6.53-10.07)	N/A	N/A
MAR PHQ-9 baseline and treatment adherence (GEE)	7.47 (6.84-8.15)	−8 (−16 to 0)	Statistical equivalence
MAR PHQ-9 baseline and treatment adherence (mixed)	7.5 (6.89-8.16)	−8 (−19 to 6)	
MAR GAD-7 baseline and treatment adherence (GEE)	7.15 (6.7-7.63)	−12 (−24 to 3)	Statistical equivalence
MAR GAD-7 baseline and treatment adherence (mixed)	7.28 (6.81-7.78)	−10 (−23 to 4)	
MAR treatment adherence (GEE)	6.91 (6.6-7.25)	−15 (−19 to −11)	Statistical equivalence
MAR treatment adherence (mixed)	7.06 (6.71-7.43)	−13 (−26 to 3)	
MAR PHQ-9 baseline (GEE)	6.57 (6.07-7.12)	−19 (−25 to −12)	Statistical equivalence
MAR PHQ-9 baseline (mixed)	6.54 (6.05-7.07)	−19 (−30 to −7)	
MAR comorbidity and education, and age (GEE)	6.31 (5.99-6.65)	−22 (−26 to −18)	Significant underestimation
MAR comorbidity and education, and age (mixed)	6.33 (6.01-6.66)	−22 (−34 to −8)	
MAR comorbidity (GEE)	6.23 (5.9-6.57)	−23 (−27 to −19)	Significant underestimation
MAR comorbidity (mixed)	6.24 (5.93-6.58)	−23 (−35 to −9)	
MAR GAD-7 baseline (GEE)	6.09 (5.82-6.37)	−25 (−28 to −21)	Significant underestimation
MAR GAD-7 baseline (mixed)	6.12 (5.85-6.4)	−25 (−36 to −10)	
MAR age (GEE)	6.03 (5.97-6.08)	−26 (−26 to −25)	Significant underestimation
MAR age (mixed)	6.07 (6.01-6.13)	−25 (−39 to −8)	
MAR Marital Status (GEE)	6 (5.83-6.17)	−26 (−28 to −24)	Significant underestimation
MAR marital Status (mixed)	6.03 (5.87-6.2)	−26 (−38 to −10)	
MAR education (GEE)	5.96 (5.88-6.04)	−27 (−28 to −26)	Significant underestimation
MAR education (mixed)	5.99 (5.91-6.07)	−26 (−40 to −9)	
MAR gender (GEE)	5.94 (5.89-6)	−27 (−27 to −26)	Significant underestimation
MAR gender (mixed)	5.98 (5.93-6.03)	−26 (−40 to −9)	

^a^Relative accuracy from observed recontacted cases following a clinical follow-up.

## Discussion

### Principal Findings

The primary aim of this study was to examine the characteristics, likely clinical outcomes, and statistical solutions that could be applied when missing cases in Web-based psychotherapy are encountered. This was done by first exploring the characteristics of missing cases within a large, naturalistic Web-based treatment sample; specifically identifying those participant characteristics that could predict the likelihood an individual would become missing following treatment, and at the same time, predict the outcomes such individual was likely to experience. In addition, this study attempted to test the suitability and accuracy of different statistical solutions for replacing missing cases (eg, adjusted and unadjusted model approximations, LOCF, and BOCF replacement strategies) through the comparison of statistically approximated outcomes against known outcomes from cases who were missing and were successfully recontacted (recontacted cases). The results were organized with three interrelated steps.

In a fundamental first step, the features of treatment adherence rates and baseline symptom severity were identified as predictors that can significantly increase the likelihood of participants to become missing at posttreatment. Together, treatment adherence and baseline symptoms explained 41% of the probability variance of missing cases status and were identified as the dominant predictor from a range of alternatives predictors initially included in the model. In this way, the first hypothesis, stating that missing cases were not occurring at random, was supported. This result demonstrated support for the first hypothesis, stating that missing cases were not occurring at random.

Critically, the variables of treatment adherence and baseline symptoms also shaped the clinical outcomes missing cases were likely to experience. Specifically, poorer treatment adherence was also associated with increased symptoms and distinct symptom outcomes. Similarly, higher pretreatment symptoms were associated with higher symptoms following treatment. This finding supported the second hypothesis and is consistent with research about the role of dosage, adherence, and treatment outcomes [37-39]. At the same time, the association of increased symptoms and missing cases is also in line with previous research, suggesting that severely depressed participants are more likely to drop out [1,2,38]; and in parallel, an association between baseline severity and increased residual symptoms at posttreatment [40]. Recognizing treatment adherence and baseline severity as variables that predict both who will become missing and their likely clinical outcomes is key for understanding the likely clinical trajectory of missing cases.

In more statistical terms, this study demonstrated that missing cases cannot be assumed to be a random portion of the overall sample (MCAR), and overlooking the specific pattern of treatment adherence and baseline severity can result in overestimation of treatment efficacy and underestimate remaining symptom. The additional comparison of proximal recontacted cases with replacement methods such as LOCF and BOCF also demonstrated significant measurement error with overestimation that is as high as 70%; consistent with previous research [41,42], indicating these methods lead to overly conservative underestimates of treatment benefits.

Finally, testing of the third hypothesis demonstrated that missing cases could be predicted with minimal error; however, only by accounting for the specific variables that influence both missing cases likelihood and clinical outcomes. Specifically, among all the available model-based approximation methods, models that adjust their estimate of clinical outcomes by treatment adherence and baseline symptom severity demonstrated acceptable statistical accuracy. Using either GEE or mixed methodologies, models that adjusted for both treatment adherence and baseline severity of symptoms resulted in prediction that were only 8% lower than actual values of recontacted cases and were considered statistically equivocal. This result can also be interpreted as a verification of the suitability of replacing missing cases through adjusted replacement strategies under conditional MAR assumption; that is, given that the approximation of missing cases outcomes resulted in minimum differences from the observed outcomes of recontacted cases, the suitability of the statistical approximation is supported. In addition, these results could be interpreted as refuting of the MNAR assumption, given that missing cases were accurately captured under conditionally adjusted models (adjusted for treatment adherence and baseline symptoms).

To our knowledge, this is the first study to use naturalistic measurement to verify whether missing psychotherapy cases conceal poorer clinical outcomes, as well as explore both the bias and underpinning causes. These findings are, however, consistent with current thinking about the potential causes of, and outcomes for, missing cases [1,2,9,20], as well as a long standing statistical requirement to take steps to identify and resolve missing cases bias [6,9,10].

The importance of recognizing key predictors of missing cases, as well as their clinical outcomes can be considerable. Missing cases in psychotherapy research are common [1,2] and can pose a fundamental challenge for measurement and interpretation of clinical effects [43]. On the basis of the present findings, researchers seeking to produce accurate and more complete estimates of treatment outcomes should consider whether missing cases in their own datasets show an association with variables such as treatment adherence and baseline treatments. If these trends are present, missing cases and their outcomes may not be random, and further steps would be needed to truly estimate the effects treatment. Although the implications missing cases pose for other aspects of clinical measurement is beyond the scope of this paper, the pattern of results demonstrated in this paper may certainly impact additional clinical measurement practices. For example, research aiming to identify clinical moderators, quantify patient risk, evaluate treatment efficacy, or make treatment comparison may certainly be impacted by missing cases patterns, such as those identified in this study, or additional patterns that could be identified through similar other research.

### Limitations and Future Directions

Although this study relied on a large clinical sample with high internal reliability, the results and conclusions drawn must be considered with several limitations. First, and foremost, the demonstration of missing cases characteristics, their approximated outcomes, and the suitability of replacing missing cases is preliminary and specific to a treatment model (iCBT) [30]. As shown by previous research [1], the proportion of missing values and clinical outcomes vary widely between trials. This variability may suggest that different clinical samples could also show both different predictors of missing cases and different outcome trajectories experienced by missing cases. However, broadly speaking, given that treatment engagement and initial depressive symptom rate commonly associated with both treatment adherence [2] and outcomes [41], these variable may reflect a critical starting point for the examination of missing cases in other Web-based psychotherapy trials, if not psychotherapy in general.

A second limitation relates to the use of recontacted cases to verify the suitability of statistical methods to replace missing cases. This sample of recontacted cases relied on a modest sample of 55 recontacted cases. Despite efforts to empirically compare recontacted cases with completely missing cases, recontacted cases can only be assumed to represent the larger group of missing cases. Albeit the uncertainty associated with recontacted cases, it is important to note that recontacted cases embody naturally occurring proximal outcomes that cannot be researched with artificial statistical studies. Given that no alternative is currently available to verify the outcomes for missing cases, recontacted cases may prove a novel future measurement proxy for missing cases as a broader group.

To address both limitations, replication of these missing patterns and research methodology in other similar treatment samples is key. It is important to note that investigating missing cases in naturalistic, clinical settings, as well as collating a sizeable group of recontacted cases is not straightforward given their rarity (eg, 55/820). However, increasingly large and standardized psychotherapy databases are becoming available [1], and these large databases may enable to similarly research methodology and exploration of predictors, outcomes and proximal measurements for missing cases.

In addition, it is important to acknowledge that this study does not pertain to exhaust the theoretical causes, or the identification of predictors that may underpin missing cases and their outcomes. Other alternative important participant variables could indeed play a role in underpinning why cases become missing and how their outcomes should be approximated. For example, the presence of a major depression diagnosis [39], credibility, or motivation [38] may lead to different rates of treatment adherence and at the same time, better capture the trajectory of missing cases in treatment. For this reason, similar future studies may consider a more direct measurement of participant engagement that may underpin their trajectory in treatment. For example, measurements of motivation, enthusiasm, clinical barriers, treatment credibility, or other clinical consideration may offer a more interruptible means to profile missing cases and their likely clinical outcomes.

Furthermore, it is important to consider that the ability to use adjusted approximation models that factor both treatment adherence and baseline symptoms may not be realistic in small samples. For example, a psychotherapy sample of 30 or less, may be underpowered, or show insufficient variance for the use of complex adjusted statistical models. For this reason, more parsimonious and more robust solutions for replacing missing cases in smaller samples should be explored. For example, methods that are less statistically demanding, such as the application of LOCF for cases who do not complete treatment, could be coupled with unadjusted (MCAR), approximation of outcome for those cases that adhere to treatment in full. This type of hybrid solution may result in a less statistically demanding strategy, which hyphenates the LOCF overly conservative approximation of outcomes, with the MCAR assumption, which is overly liberal as a method that underestimates symptom outcomes. Such solutions are beyond the scope of this paper; however, the application of corrective missing cases methods for small samples may be key for psychotherapy trials such as pilots and small RCTs.

Finally, it is important to note that results of this study imply that within Web-based psychotherapeutic interventions such as CBT-based interventions, the role of adherence and baseline symptoms could likely be important and implicit. Recognizing such patterns can lead to clearer understanding of missing cases, the assumptions that can be made about missing cases, and a more accurate consideration of their outcomes. Although these results should be considered as possible fundamental pattern in the application of any statistical replacement strategy, it important to note that this study does not advocate the use of any one statistical approach over another as means for handling missing cases. Rather, this study intended to explore the implicit characteristics that influence Web-based psychotherapy cases and suggest those measurement considerations that would likely improve the application of missing cases strategies.

In summary, this research aimed to create a more concrete awareness of missing cases and ways to handle missing cases in Web-based psychotherapeutic trials. Using concrete and transparent statistical modeling, this research demonstrated that missing cases can occur systematically and with clinical outcomes that are dissimilar to the outcomes of those individuals who are surveyed following treatment. This study also offered (1) a research design framework that can concretely quantify the outcome bias associated with naturalistically occurring missing cases, (2) highlight important predictors that explain both missing cases and their outcomes, and (3) suggest a naturalistic benchmark (recontacted cases) that could be conditionally used for quantifying the outcomes for missing cases and verifying the suitability of various statistical solutions that approximate missing cases. Together, all three aspects of characteristics, bias in outcomes, and methods to resolve the bias in outcomes should be considered preliminary and pendent on future replication.

## Abbreviations

BOCF: baseline observation carried forward
CBT: cognitive behavioral therapy
GEE: generalized estimating equation
GAD-7: Generalized Anxiety Disorder Scale 7-item
iCBT: Internet cognitive behavioral therapy
LOCF: last observation carried forward
MAR: missing at random
MCAR: missing completely at random
MNAR: missing not at random
MUM: Macquarie University Web-based Model
RCT: randomized controlled trial
PHQ-9: Patient Health Questionnaire 9-item
